# Trends in incidence, mortality, and survival for kidney cancer in Canada, 1986–2007

**DOI:** 10.1007/s10552-014-0427-x

**Published:** 2014-07-18

**Authors:** Prithwish De, Michael C. Otterstatter, Robert Semenciw, Larry F. Ellison, Loraine D. Marrett, Dagny Dryer

**Affiliations:** 1 Cancer Control Policy, Canadian Cancer Society, 55 St Clair Ave West, Suite 300, Toronto, Ontario Canada; 2 Dalla Lana School of Public Health, University of Toronto, Toronto, Canada; 3 BC Centre for Disease Control, Vancouver, Canada; 4 Centre for Chronic Disease Prevention, Public Health Agency of Canada, Ottawa, Ontario Canada; 5 Statistics Canada, Ottawa, Ontario Canada; 6 Cancer Care Ontario, Toronto, Canada; 7 PEI Cancer Treatment Centre, Charlottetown, Canada

**Keywords:** Kidney cancer, Renal cell carcinoma, Trends, Risk factors, Canada

## Abstract

**Purpose:**

Kidney cancer is one of the fastest rising cancers worldwide. We aimed to examine the trends in incidence, mortality, and survival for this cancer in Canada.

**Methods:**

Incidence data for kidney cancer for 1986–2010 were from the Canadian Cancer Registry and the National Cancer Incidence Reporting System. These data were only available up to 2007 for the province of Quebec and consequently for the same year nationally, for Canada. Mortality data for 1986–2009 were from the Canadian Vital Statistics Death Database. Changes in age-standardized rates were analyzed by Joinpoint regression. Incidence rates were projected to 2025 using a Nordpred age-period-cohort model. Five-year relative survival ratios (RSR) were analyzed for 2004–2008 and earlier periods.

**Results:**

Between 1986 and 2007, the age-standardized incidence rate (ASIR) per 100,000 rose from 13.4 to 17.9 in males and 7.7 to 10.3 in females. Annual increases in ASIR were greatest for age groups <65 years (males) and ≥65 years (females). The ASIRs increased significantly over time in both sexes for renal cell carcinoma (RCC) but not for other kidney cancer types. RCC rates are projected to increase until at least 2025. Mortality rates decreased only slightly in each sex since 1986 (0.4 %/year in males; 0.8 %/year in females). The 5-year RSR for kidney cancer was 68 % but differed largely by morphology and age, and has increased slightly over time.

**Conclusions:**

The incidence rate of kidney cancer in Canada has risen since at least 1986, led largely by RCC. Increasing detection of incidental tumors, and growing obesity and hypertension rates are possible factors associated with this increase. Greater prevention of modifiable risk factors for kidney cancer is needed.

## Background

Kidney cancer will be responsible for an estimated 6,000 new cases and 1,750 deaths in Canada in 2014, making it the eighth most common cancer and the twelfth leading cause of cancer death [1]. In developed countries, kidney cancer represents approximately 2–4 % of all new cancers, with incidence rates ranging from 7 to 22 per 100,000 among males and 4 to 13 per 100,000 among females [2, 3]. Incidence rates are higher in developed countries than in the developing world [4].

Historically, cancers of the renal pelvis were grouped with those of the renal parenchyma when referring to kidney cancer. However, it is now recognized that the former are essentially urothelial (bladder) cancers [5]. Using this definition, approximately 90 % of all kidney cancers in Canadian adults occur in individuals 45 years of age and older [6]. The most frequent morphological type is renal cell carcinoma (RCC), which represents about 80 % of all kidney cancers in Canada [6]. The remainder represents unspecified malignant neoplasms (11.0 %) and carcinomas (1.8 %), transitional and squamous cell carcinomas (2.0 %), and other specified malignant neoplasms (0.7 %) and carcinomas (0.7 %). RCC consists mostly of two histological types: clear cell carcinoma (>80 %) and papillary (10 %) [7].

Established risk factors for kidney cancer are smoking, obesity, hypertension, inherited genetic conditions (e.g., Von Hippel-Lindau syndrome), end-stage kidney disease, having a first-degree relative with kidney cancer, and a birth defect known as horseshoe kidney [8]. Possible risk factors include exposure to radiation, arsenic in drinking water, and some occupational exposures. Among occupational exposures, trichloroethylene (TCE), a chemical used in dry cleaning, has been most commonly implicated in kidney cancer risk [9, 10] and appears to be more strongly associated with clear cell RCC [11]. Certain medications may have a role in increasing the risk of kidney cancer, including acetaminophen and non-aspirin NSAIDs, but there is little consistency for this association [12]. On the contrary, moderate alcohol consumption appears to be associated with a reduction in risk of RCC [13, 14].

Given that known genetic conditions explain up to 5 % of RCC cases [15], increases in incidence rates may be explained by improvements in detection as well as lifestyle and behavioral causes. Of the lifestyle factors, tobacco use, excess body weight, and uncontrolled blood pressure are the most important modifiable risk factors at a population level.

Several countries have reported rising incidence rates of kidney cancer [2, 3, 7], particularly for RCC, but a detailed analysis of such trends in Canada has not yet been performed. Therefore, the objectives of this study were to analyze the long-term trends in incidence and mortality, examine survival, and calculate projected incidence rates up to 2025 for kidney cancer in Canada.

## Methods

### Data sources

Incidence data for 1986–1991 were obtained from the National Cancer Incidence and Reporting System and for 1992–2010 from its successor, the Canadian Cancer Registry [16].

Each provincial and territorial cancer registry supplies data on cancer patients and tumors in a standard format. Subsequent changes to registry data due to errors or omissions found in the data are also transmitted to Statistics Canada. Each provincial and territorial cancer registry has the ability to add, update, and delete records. A series of core edits and an internal record linkage process to identify duplicate records are used to build and maintain the Canadian Cancer Registry [16].

Kidney cancer cases were defined based on the International Classification of Diseases for Oncology, Third Edition (ICD-O-3) [17] as topography C64.9 (kidney, not otherwise stated) excluding morphologies 9050–9055, 9140, and 9590–9989. Cancers of the renal pelvis (C65.9) were excluded because these are now recognized as being urothelial cancers [5]. By way of comparison, for 2003–2007, the incidence is reduced from 21,335 to 20,052 when renal pelvis is excluded from our data. For data prior to 1992 from the National Cancer Incidence and Reporting System, equivalent ICD-9 site codes [18] and ICD-O-1 histology codes [19] were used.

Cancers of the renal parenchyma were examined according to morphology, as RCC or “other.” Children under 15 years of age were excluded from our analysis because the predominant type of renal cancer that develops in this group is nephroblastoma.

Mortality data were from the Canadian Vital Statistics Death Database [20] and were selected based on classification C64 used in the International Classification of Disease, version 10 [21]. Population estimates, which were used to derive rates, are from Statistics Canada’s Demographic Estimates Compendium 2012 [22].

### Statistical analysis

#### Incidence and mortality rates

Incidence data for one Canadian province (i.e., Quebec) were only available up to 2007 despite data for all other provinces being available to 2010. As a result, we restricted our analysis of national incidence rates for Canada up to 2007 but examined rates for provinces up to their latest available year of data. Cancer mortality data, on the other hand, were available up to 2009 for all regions. Canadian territories (Yukon, Northwest Territories, Nunavut) were excluded from region-specific analyses presented in this paper because of the small number of annual new cases and deaths in these jurisdictions. Data from the territories were, however, included in the analyses of national rates for Canada.

Age-standardized incidence rates (ASIR) for 1986–2007 for Canada (and up to 2010 for all provinces except Quebec) and age-standardized mortality rates (ASMR) for 1986–2009 for Canada and all provinces were derived using the direct method and standardized to the 1991 Canadian population [1]. Trends over time were analyzed by calculating the annual percent change (APC) and average annual percent change (AAPC) in age-standardized rates using Joinpoint regression [23]. The minimum number of observations from a joinpoint to either end of the data and between joinpoints was set to five.

#### Incidence rate projections

The Nordpred Power5 age-period-cohort model was used to project Canada incidence rates for five-year periods from 2008–2012 to 2023–2027. Nordpred is based on an age-period-cohort Poisson regression model but has enhancements that overcome difficulties in the standard Poisson model and improve projection accuracy [24, 25]. Future values are estimated by projecting forward the model’s linear time trend, with successive reductions in each period, while fixing the period and cohort effects. Nordpred default settings were used for all projections. A detailed description of the projection model and methods can be found elsewhere [26].

#### Survival

Relative survival analyses were based on a publicly available algorithm [27] to which minor adaptations were made. Expected survival proportions were derived from both published [28] and unpublished annual sex-specific complete provincial life tables. The focus of this analysis was on all primary cancer cases aged 15–99 at diagnosis. Mortality follow-up through December 31, 2008, was determined by record linkage of the Canadian Cancer Registry to the Canadian Vital Statistics Deaths database and from information reported by provincial and territorial cancer registries. Data from the province of Quebec were excluded for survival analyses only because the method of ascertaining the date of diagnosis of cancer cases in this province differed from that of other provincial cancer registries and because of issues in correctly ascertaining the vital status of cases diagnosed in Quebec within the Canadian Cancer Registry. Data for the Canadian territories (Yukon, Northwest Territories, Nunavut) and the province of Prince Edward Island are not presented separately because of the small number of cases, which do not allow for precise estimates to be calculated. Five-year relative survival ratio (RSR) estimates were derived using the period method for 2004–2008 and the cohort method for earlier time periods of 1992–1996 and 1998–2002. Further detail on the survival methodology is provided elsewhere [29].

## Results

Between 1986 and 2007, the ASIR of kidney cancer in Canada rose from 13.4 to 17.9 per 100,000 in males and 7.7 to 10.3 per 100,000 in females (Table 1), representing an AAPC of 1.2 % per year in males and 0.9 % per year in females over this period. The greatest increase in incidence rate in males occurred between 1986 and 1990, when the APC was 3.5 %. A second period of significant increase in males occurred from 1998 to 2007, when the APC was 1.7 %. In females, the rate rose significantly by 2.0 % per year from 1998 to 2007.

**Table 1:** Annual percent change and average annual percent change for incidence and mortality rates for kidney cancer (excluding renal pelvis), ages 15 + , Canada, 1986–2007

	Age-standardized rate* (per 100,000)		Trend 1		Trend 2		Trend 3		Full study period	
	1986	2007	Years	APC (95 % CI)	Years	APC (95 % CI)	Years	APC (95 % CI)	Years	AAPC (95 % CI)
Incidence	(*n* = 2015)	(*n* = 4,490)								
Males	13.4	17.9	1986–1990	3.5^†^ (0.9, 6.2)	1990–1998	−0.4 (–1.4, 0.6)	1998–2007	1.7^†^ (1.1, 2.3)	1986–2007	1.2^†^ (0.6, 1.8)
Females	7.7	10.3	1986–1998	0.1 (–0.6, 0.9)	1998–2007	2.0^†^ (1.0, 3.0)	−	−	1986–2007	0.9^†^ (0.4, 1.5)
Mortality	(*n* = 923)	(*n* = 1,445)								
Males	6.6	6.2	1986–2007	−0.4^†^ (−0.7, −0.2)	–	–	–	–	1986–2007	−0.4^†^ (−0.7, −0.2)
Females	3.2	2.8	1986–2007	−0.8^†^ (−1.0,−0.5)	–	–	–	–	1986-2007	−0.8^†^ (−1.0,− 0.5)

Between 1986 and 2007, there were no change points for the male and female mortality rates. As a result, the AAPC is equivalent to the APC over the specified time period

*APC* annual percent change, *AAPC* average annual percent change, *CI* confidence interval

* Rates are age-standardized to the 1991 Canadian population

^†^Statistically significantly different from zero (two-sided *p* < 0.05)

Age-standardized mortality rates were less than half of the corresponding incidence rates and declined only slightly since 1986 (Table 1). The ASMR declined by 0.4 % per year in males and 0.8 % per year in females.

### Trends by age and sex

**Fig. 1:**
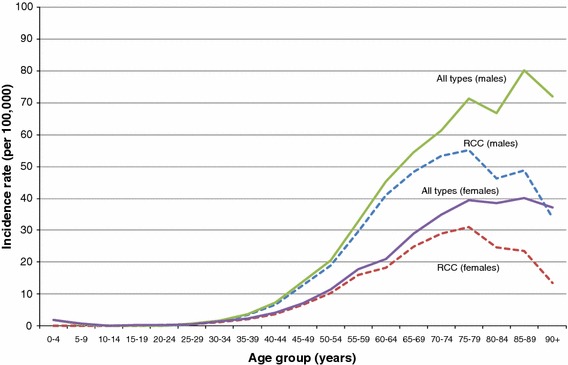
Age-specific incidence rates of renal cell carcinoma (RCC) and all kidney cancer subtypes (including RCC but excluding renal pelvis), Canada, 2003–2007

The age-specific incidence rate of RCC generally rose quickly after age 50 for both sexes and followed the trend for all kidney cancer types combined. However, this rate dropped sharply after the 75–79 age group (Fig. 1). The “unknown” histology group was responsible for the continuing increasing trend in the older age groups beyond 75 years.

**Fig. 2:**
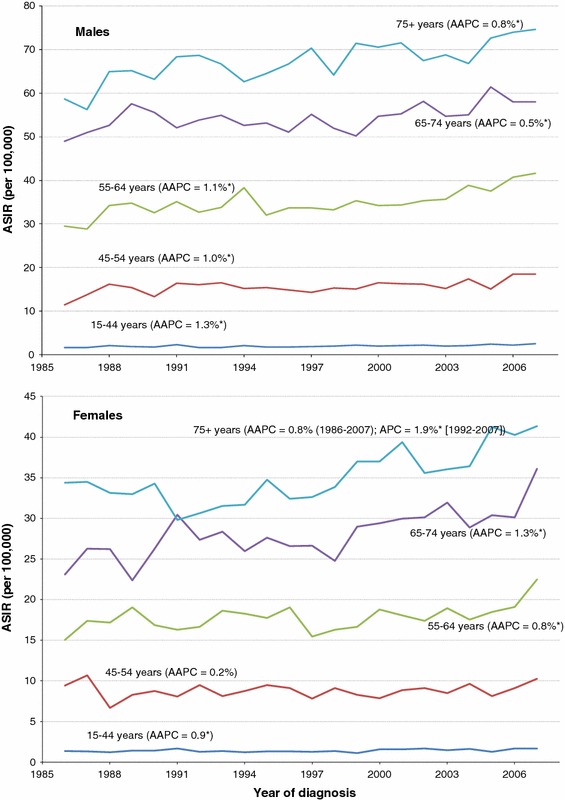
Age-standardized incidence rates (ASIR) of kidney cancer (excluding renal pelvis) by age group and sex, Canada, 1986–2007. *AAPC* average annual percent change; *APC* annual percent change. Change point observed only in ASIR for females aged 75+ years (in 1992). * statistically significantly different from zero (two-sided *p* < 0.05)

**Fig. 3:**
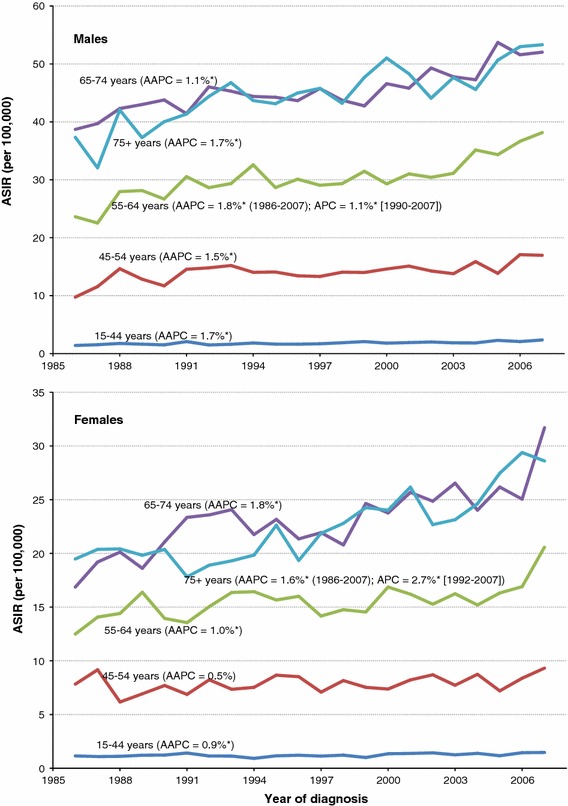
Age-standardized incidence rates (ASIR) of RCC by age group and sex, Canada, 1986–2007. *RCC* renal cell carcinoma; *AAPC* average annual percent change; *APC* annual percent change. * statistically significantly different from zero (two-sided *p* < 0.05)

Among males, the ASIR of kidney cancer has increased over time across all age groups (Fig. 2), with the biggest increases in those under age 65. In contrast, incidence rates have increased most among females over the age of 65. When RCC was examined separately by age group, similar trends were observed, with higher AAPC in most age groups (Fig. 3). In particular, the AAPC was more than double in the two oldest age groups in males (65–74 years and 75 + years).

Mortality from kidney cancer (data not shown) has generally declined across all age groups, with the strongest declines among those aged 15–44 years (males: –2.5 % per year; females –3.1 % per year). The only exceptions were an increase in the mortality rate among males aged ≥75 years (0.7 % per year) and stable mortality rates among females aged ≥65 years.

**Fig. 4:**
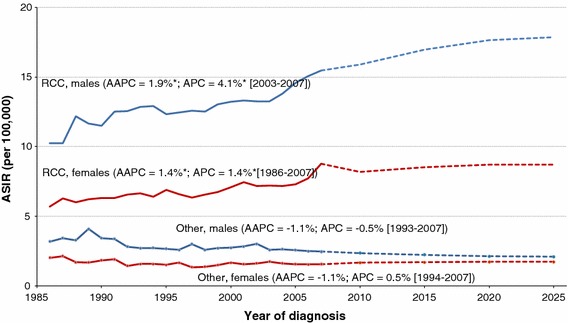
Age-standardized incidence rates (ASIR) for RCC and other kidney subtypes (excluding renal pelvis) by sex in Canada for 1986–2007 and projections for 2008–2025. *RCC* renal cell carcinoma; *AAPC* average annual percent change; *APC* annual percent change. ‘Other’ includes all cases that are not RCC. * statistically significantly different from zero (two-sided *p* < 0.05)

Unlike for other kidney cancer subtypes, the ASIR has increased significantly since 1986 for RCC in both males (1.9 % per year) and females (1.4 % per year) of all ages (Fig. 4). Among males, the ASIR for RCC has risen by 4.1 % per year between 2003 and 2007. Projections for the ASIR of RCC in males and females show an upward trajectory until at least 2025, reaching rates of 17.9 per 100,000 in males and 8.7 per 100,000 for females. Little or no change for other kidney cancer subtypes is expected.

### Trends by province

Based on data for 1986–2010 (up to 2007 for Quebec), the ASIR was nearly two times higher among males than females in most provinces (Table 2). Provincial ASIRs were generally higher in eastern Canada (i.e., New Brunswick, Newfoundland & Labrador, Nova Scotia, and Prince Edward Island).

**Table 2:** Annual percent change in age-standardized incidence rates for kidney cancer (excluding renal pelvis), ages 15 + , by sex in Canada and provinces*

	Males			Females		
	ASIR (per 100,000)	APC (95 % CI)	Changepoint^†^	ASIR (per 100,000)	APC (95 % CI)	Changepoint^†^
Canada	17.9	1.7^‡^ (1.1,2.3)	1998	10.3	2.0^‡^ (1.0,3.0)	1998
Province						
British Columbia	11.0	−0.4 (−0.8,0.0)		5.2	−0.4 (−0.9,0.1)	
Alberta	15.6	0.4 (−0.2,0.9)		8.6	0.2 (−0.4,0.7)	
Saskatchewan	17.9	0.6 (−0.2,1.4)		9.8	1.0^‡^ (0.1,1.8)	
Manitoba	23.0	1.8^‡^ (1.0,2.6)		10.0	−0.5 (−2.6,1.7)	2000
Ontario	17.6	2.9^‡^ (0.6,5.2)	2003	9.0	1.4^‡^ (1.0,1.8)	
Quebec	18.4	1.0^‡^ (0.7,1.3)		10.2	2.0^‡^ (1.2,2.8)	1997
New Brunswick	28.9	2.6^‡^ (1.6,3.5)		10.4	1.2^‡^ (0.1,2.3)	
Nova Scotia	22.1	1.6^‡^ (0.9,2.2)		13.5	1.7^‡^ (1.0,2.4)	
Prince Edward Island	22.1	1.2 (−0.5,2.9)		10.6	1.9 (−0.1,3.9)	
Newfoundland & Labrador	23.7	2.7^‡^ (1.7,3.6)		13.2	1.7 (−0.1,3.5)	

*APC* annual percent change, *ASIR* age-standardized incidence rate, *CI* confidence interval

* Canada and Quebec ASIR is for 2007 and APC for 1986-2007; ASIR for all other provinces is for 2010 and APC for 1986–2010

^†^Change point refers to the year in which the APC changed. Therefore, the associated APC refers to annual percent change from that point to the year 2010 (or 2007 for Canada and Quebec). If no change point is indicated, then the APC refers to the average annual percent change (AAPC) over the period of 1986–2010 (or 1986–2007 for Canada and Quebec)

^‡^Statistically significantly different from zero (two-sided *p* < 0.05)

When comparing the change in the ASIR over time, the largest APCs for males over the period of 1986–2010 were observed in Ontario (since 2003), New Brunswick, and Newfoundland & Labrador. In females, the largest APCs were in Quebec (since 1997). With the exception of males in Manitoba, APCs were lowest in western Canada (i.e., British Columbia, Alberta, and Saskatchewan) in both sexes.

### Five-year relative survival

Individuals diagnosed with kidney cancer in Canada in 2004–2008 were estimated to have 68 % chance of living at least five more years after diagnosis as compared to similar people in the general population (Table 3). The five-year RSR for those with RCC of the kidney was estimated at 72 %—considerably higher than for cases diagnosed with another or unknown histology.

**Table 3:** Estimated five-year relative survival ratios (%) for kidney cancer (excluding renal pelvis) by time period, histology, sex, age group, and province in Canada*, 1992-2008

	Five-year RSR (95 % CI) unadjusted for age	Age-standardized five-year RSR (95 % CI)		
	2004–2008	1992–1996	1998–2002	2004–2008
Histology				
All kidney cancers	68 (67–69)	60 (59–61)	64 (63–65)	67 (66–68)
Renal cell carcinoma	72 (71–73)	65 (63–66)	68 (67–69)	71 (70–72)
Other & unspecified	43 (41–46)	42 (39–45)	42 (39–44)	48 (45–50)
Sex				
Males	67 (66–68)	59 (58–61)	62 (61–64)	66 (65–67)
Females	69 (68–71)	62 (61–64)	66 (65–68)	69 (68–70)
Age group^†^				
15–44	85 (83–87)	79 (76–82)	84 (81–86)	85 (83–87)
45–54	77 (75–78)	69 (66–71)	74 (72–76)	77 (75–78)
55–64	71 (70–73)	63 (61–66)	68 (66–70)	71 (70–73)
65–74	66 (64–68)	60 (58–62)	62 (60–64)	66 (64–68)
75–99	54 (51–56)	48 (45–51)	50 (47–53)	54 (51–56)
Province				
British Columbia	62 (59–64)	58 (56–61)	57 (55–60)	62 (60–64)
Alberta	65 (62–67)	59 (56–62)	63 (60–65)	63 (61–66)
Saskatchewan	63 (59–68)	52 (47–57)	61 (56–65)	64 (60–68)
Manitoba	58 (54–62)	53 (49–57)	59 (55–63)	58 (55–62)
Ontario	73 (71–74)	63 (62–65)	67 (66–69)	72 (70–73)
New Brunswick	68 (64–73)	55 (50–60)	64 (59–68)	66 (62–70)
Nova Scotia	67 (63–71)	64 (59–69)	62 (58–66)	67 (63–71)

*RSR* relative survival ratio, *CI* confidence interval

* Excludes the province of Quebec. Data from the province of Quebec were excluded because the method of ascertaining the date of diagnosis of cancer cases in this province differed from that of other provincial cancer registries and because of issues in correctly ascertaining the vital status of cases diagnosed in Quebec within the Canadian Cancer Registry. Data for the province of Newfoundland and Labrador are not shown as they are artifactually high

^†^Age group-specific results were not age-standardized

Estimated five-year RSRs were slightly higher among females than males and progressively declined with advancing age from 85 % among those aged 15–44 years at diagnosis to 54 % among those aged 75–99. Age-standardized five-year RSR ranged from a high of 73 % among those diagnosed in the province of Ontario to a low of 58 % in Manitoba. In all other provinces, RSRs were generally in the mid-60 s.

Absolute increases in survival from 1992–1996 to 2004–2008 by sex, age group, and histology were consistent with the increase observed overall for kidney cancer (7 percentage points). The largest provincial increases in RSR over time occurred in Saskatchewan (12 %) and New Brunswick (11 %).

## Discussion

### Incidence

In Canada, kidney cancer incidence rates have been increasing since at least 1986 and, according to an earlier analysis [30], have nearly doubled since 1970. The greater rate of increase in males under age 65 and in females over 65 years possibly reflects the prevalence of obesity in Canada, whereby self-reported obesity is markedly more prevalent among males up to the age group of 55–64 but is more prevalent in females after the age of 75 years [31]. The plateauing of incidence rates for RCC followed by a drop in rates after age 75 in Canadian males and females (Fig. 1) may be due to less rigorous diagnostic testing and microscopic confirmation of cases compared to all other types of kidney cancers in this age group [32].

If historical trends continue, rates of RCC in Canada are projected to rise by about 15 % among males by 2025 (Fig. 4), but increase only marginally among females. Similar patterns of increase are expected in most parts of the world [33]. Aside from RCC, the incidence rate for other histological types has been relatively stable in Canada in recent years, and little change is expected over the next decade. Our projected future rates of kidney cancer are estimates based on the assumption that historical trends will continue. Actual future values may differ as a result of, for example, changes in prevention programs and the prevalence of key risk factors.

The rising incidence of kidney cancer is consistent with trends observed by others over a similar time period [32, 34–36]. The increasing use of imaging technologies (e.g., ultrasonography, computed tomography, magnetic resonance imaging) has likely resulted in greater incidental detection of kidney cancer [37, 38], especially of smaller tumors [32, 39]. However, such technological advancements cannot entirely account for the upward trends [35] as incidence rates have continued to increase despite the fact that improvements in detection occurred primarily during the late 1980s to early 1990s. Despite increased RCC rates associated with period-related factors, birth cohorts since the 1960s in the USA also show rapidly increasing incidence rates compared to earlier cohorts [40]. This suggests that risk factors and exposures unique to recent generations account to some degree for the rising incidence of RCC. However, the relative contribution of changes in risk factor prevalence in comparison with changes in diagnostic practices remains unclear.

### Risk factors

Several modifiable risk factors may be contributing to the upward incidence trend in Canada, including obesity, hypertension, and smoking. However, the relative contribution of each of the above risk factors to the temporal trend in kidney cancer is difficult to ascertain given the possible confounding effect of increasing detection of this disease [41].

The prevalence of obesity has been increasing in Canada over the past 25 years [42], a trend that has mirrored the rise in kidney cancer rates. A positive association between body mass index (BMI) and kidney cancer has been previously reported in Canada, with the greatest risk in the most obese [43, 44]. Obesity is attributed to 30–40 % of kidney cancer cases [7] and it is strongly linked to clear cell RCC in a dose–response fashion [45, 46].

Hypertension is a well-established risk factor for kidney cancer, independent of obesity, and elevates the risk of RCC in a dose-dependent manner [7]. In Canada, there has been a rising trend in the prevalence of measured hypertension since at least the mid-1980s [47, 48]. Even well-controlled hypertension is associated with an elevated risk of kidney cancer, with the relative risk of cancer being only slightly lower than among people with poorly controlled blood pressure [49].

Smoking is a well-established risk factor for RCC [50], and despite its declining prevalence in Canada, likely continues to contribute to new RCC cases. Smoking has been implicated as a causal factor in up to 20 % of kidney cancer cases [15], and the association with smoking appears to be stronger for cancers of the renal pelvis than the renal parenchyma [51]. Among active smokers, the risk of kidney cancer increases with the number of cigarettes smoked per day [52], with at least a 50 % increased risk of kidney cancer [53]. Moreover, there is evidence that exposure to second-hand smoke also increases risk, with longer duration and multiple sources of exposure leading to higher risk [54, 55].

Provincial variations in kidney cancer incidence rates likely reflect regional differences in the prevalence of risk factors. First, the higher incidence rates of kidney cancer in Prince Edward Island, Nova Scotia, New Brunswick, Quebec, Saskatchewan, and Manitoba could partly be explained by the higher prevalence of smoking in these provinces compared to other parts of Canada [56]. Second, adult obesity rates have tended to be higher in eastern Canada (i.e., provinces of Nova Scotia, New Brunswick, Prince Edward Island, and Newfoundland & Labrador) and two Territories (Northwest Territories and Nunavut) but have generally increased across the country between 2000 and 2011 [57]. Third, in 2006–07, the prevalence of hypertension was highest in eastern Canada, followed by the provinces of Saskatchewan, Manitoba, and Ontario. Finally, exposure to inorganic arsenic in drinking water and food (a possible cause of kidney cancer [58]) also varies across the country, with greatest amounts of geologic deposits in the Yukon, northern British Columbia, Nunavut Islands, eastern Canada, and a few areas in southern Ontario [59].

### Mortality

The mortality rates for both sexes in 2007 have decreased only slightly compared to 1986. The small decline is consistent with other studies in Canada [34] and in Europe [2], where the advent of improved diagnostics in the early 1990s, a greater understanding of the molecular biology of the disease, surgical advances, and systemic chemotherapies for the disease have all likely played a role.

### Survival

The diverging trends in incidence and mortality rates suggest an improvement in survival. The age-standardized five-year RSR in Canada has increased by 7 % points (60 % to 67 %) from 1992/1996 to 2004/2008. Part of this improvement may be due to the identification of an increasing proportion of early-stage tumors with better prognosis [60], which has been observed by several authors [32, 41].

In the province of Manitoba in 2010, 50.0 % of new kidney cancer diagnoses were stage I and 24.7 % were stage IV [61], suggesting a potentially important role for the early detection of this cancer. Although stage IV (metastatic disease) is fatal for the vast majority of people, individuals with stage I are expected to have a good prognosis [62], with data from the USA showing that the five-year relative survival at localized stage to be 91.7 % [63].

The overall five-year RSR of kidney cancer of 68 % (unadjusted for age) is close to the average survival of 63 % for all cancers combined in Canada [1]. The RSR depended strongly on morphology (with higher survival for RCC versus other types), and age (with higher survival for younger ages). In comparison with Canada, five-year relative survival from kidney cancer appears to be somewhat lower across Europe [64] and slightly higher in the USA [63].

### Implications

While the role of increasing detection of incidental tumors cannot be ruled out, research to date also suggests an important role for lifestyle risk factors in the etiology of kidney cancer. Obesity is identified as an important target for intervention [4, 58,] with some research suggesting that obesity at any stage of life affects the risk of RCC [65]. Managing excess weight may not only help prevent kidney cancer but can also have benefits for other cancers implicated by excess weight, such as cancers of the liver, thyroid, and esophageal adenocarcinoma [66–68].

Some research [69, 70] suggests that controlling blood pressure can help lower RCC risk. In addition, quitting smoking may lower the risk of kidney cancer for smokers and have collateral benefits for non-smokers exposed to environmental tobacco smoke. Quitting smoking for more than 10 years can produce the greatest reduction in kidney cancer risk, and this benefit increases with longer periods of cessation [55]. However, given the stronger association of smoking with cancers of the renal pelvis compared to RCC, reductions in smoking rates may have only a marginal impact on overall kidney cancer trends.

Although screening for kidney cancer is advised for patients and their families who have genetic conditions such as Von Hippel-Lindau syndrome, there is currently little evidence to support the effectiveness of routine screening for RCC in the general population because of the low prevalence of the disease and the high costs of imaging [71].

## Conclusion

The kidney cancer incidence rate in Canada has been rising since at least 1986, led largely by the upward trend in RCC. The increase in RCC is projected to continue into the future. Detection of incidental tumors, obesity, and hypertension, and possibly smoking, have likely contributed to the observed increase. While the kidney cancer mortality rates have been decreasing and survival increasing during the most recent decade, five-year relative survival remains moderate compared to the survival for all cancers combined in Canada.

## Abbreviations

AAPC: Average annual percent change
APC: Annual percent change
ASIR: Age-standardized incidence rate
RSR: Relative survival ratio

## References

[1] Canadian Cancer Society’s Advisory Committee on Cancer Statistics (2014) Canadian cancer statistics 2014. Canadian Cancer Society, Toronto

[2] Levi F, Ferlay J, Galeone C, Lucchini F, Negri E, Boyle P (2008). The changing pattern of kidney cancer incidence and mortality in Europe. BJU Int.

[3] Siegel R, Ma J, Zou Z, Jemal A (2014). Cancer statistics, 2014. CA Cancer J Clin.

[4] Weikert S, Ljungberg B (2010). Contemporary epidemiology of renal cell carcinoma: perspectives of primary prevention. World J Urol.

[5] Scelo G, Brennan P (2007). The epidemiology of bladder and kidney cancer. Nat Clin Pract Urol.

[6] Canadian Cancer Society’s Steering Committee (2010) Canadian Cancer Statistics 2010. Canadian Cancer Society, Toronto

[7] Chow WH, Dong LM, Devesa SS (2010). Epidemiology and risk factors for kidney cancer. Nat Rev Urol.

[8] Cho E, Lindblad P, Adami H-O, Adami H-O, Hunter D, Trichopoulos D (2008). Kidney cancer. Textbook of cancer epidemiology.

[9] Karami S, Lan Q, Rothman N, Stewart PA, Lee KM, Vermeulen R (2012). Occupational trichloroethylene exposure and kidney cancer risk: a meta-analysis. Occup Environ Med.

[10] Chiu WA, Jinot J, Scott CS, Makris SL, Cooper GS, Dzubow RC (2013). Human health effects of trichloroethylene: key findings and scientific issues. Environ Health Perspect.

[11] Karami S, Colt JS, Schwartz K, Davis FG, Ruterbusch JJ, Munuo SS (2012). A case–control study of occupation/industry and renal cell carcinoma risk. BMC Cancer.

[12] Choueiri TK, Je Y, Cho E (2013) Analgesic use and the risk of kidney cancer: a meta-analysis of epidemiologic studies. Int J Cancer 10.1002/ijc.28093 PMC3815746 23400756

[13] Song DY, Song S, Song Y, Lee JE (2012). Alcohol intake and renal cell cancer risk: a meta-analysis. Br J Cancer.

[14] Bellocco R, Pasquali E, Rota M, Bagnardi V, Tramacere I, Scotti L (2012). Alcohol drinking and risk of renal cell carcinoma: results of a meta-analysis. Ann Oncol.

[15] Lipworth L, Tarone RE, McLaughlin JK (2006). The epidemiology of renal cell carcinoma. J Urol.

[16] Statistics Canada (2013) Canadian cancer registry. http://www23.statcan.gc.ca/imdb/p2SV.pl?Function=getSurvey&SDDS=3207&lang=en&db=imdb&adm=8&dis=2. Accessed 19 Nov 2013

[17] Fritz A, Percy C, Jack A, Shanmugaratnam K, Sobin L, Parkin D (2000). International classification of disease for oncology.

[18] World Health Organization (1977) International classification of diseases, ninth revision. Volumes 1 and 2. Geneva, Switzerland: World Health Organization

[19] World Health Organization (1976). International classification of diseases for oncology.

[20] Statistics Canada (2012). Causes of death, 2009.

[21] World Health Organization (1992). International statistical classification of diseases and related health problems.

[22] Statistics Canada (2012). Demographic estimates compendium 2012.

[23] National Cancer Institute Statistical research and applications branch (2013) Joinpoint regression program. Version 4.0.3. http://srab.cancer.gov/joinpoint

[24] Fekjær H, Møller B (2011) Nordpred software package. www.kreftregisteret.no/software/nordpred. Accessed 30 Dec 2011

[25] Moller B, Fekjaer H, Hakulinen T, Sigvaldason H, Storm HH, Talback M (2003). Prediction of cancer incidence in the Nordic countries: empirical comparison of different approaches. Stat Med.

[26] Moller B, Fekjaer H, Hakulinen T, Tryggvadottir L, Storm HH, Talback M (2002). Prediction of cancer incidence in the Nordic countries up to the year 2020. Eur J Cancer Prev.

[27] Dickman P (2012) Estimating and modeling relative survival using SAS. www.pauldickman.com/rsmodel/sas_colon/. Accessed 1 May 2012

[28] Statistics Canada (2013) Life tables, Canada, Provinces and Territories, Catalogue no. 84–537-X. Minister of Industry, Ottawa. http://www5.statcan.gc.ca/bsolc/olc-cel/olc-cel?catno=84-537-XWE&lang=eng

[29] Statistics Canada (2012) Cancer survival statistics, 1992–2003. Catalogue no. 82–226-X. Minister of Industry, Ottawa. http://www.statcan.gc.ca/pub/82-226-x/82-226-x2012001-eng.pdf

[30] Kachuri L, De P, Ellison LF, Semenciw R, Advisory Committee on Canadian Cancer Statistics (2013). Cancer incidence, mortality and survival trends in Canada, 1970–2007. Chronic Dis Inj Can.

[31] Public Health Agency of Canada (2011). Obesity in Canada: a joint report from the Public Health Agency of Canada and the Canadian Institute for Health Information.

[32] Chow WH, Devesa SS (2008). Contemporary epidemiology of renal cell cancer. Cancer J.

[33] Birkhäuser F, Kroeger N, Pantuck A (2013) Etiology of renal cell carcinoma: incidence, demographics, and environmental factors. In: Campbell SC, Rini BI (eds). Current clinical urology: Humana Press pp 3–22

[34] Liu S, Semenciw R, Morrison H, Schanzer D, Mao Y (1997). Kidney cancer in Canada: the rapidly increasing incidence of adenocarcinoma in adults and seniors. Can J Public Health.

[35] Chow WH, Devesa SS, Warren JL, Fraumeni JF (1999). Rising incidence of renal cell cancer in the United States. JAMA.

[36] Mathew A, Devesa SS, Fraumeni JF, Chow WH (2002). Global increases in kidney cancer incidence, 1973–1992. Eur J Cancer Prev.

[37] Murai M, Oya M (2004). Renal cell carcinoma: etiology, incidence and epidemiology. Curr Opin Urol.

[38] Patard JJ (2009). Incidental renal tumours. Curr Opin Urol.

[39] Gill IS, Aron M, Gervais DA, Jewett MA (2010). Clinical practice. small renal mass. N Engl J Med.

[40] Tyson MD, Humphreys MR, Parker AS, Thiel DD, Joseph RW, Andrews PE (2013). Age-period-cohort analysis of renal cell carcinoma in United States adults. Urology.

[41] Sun M, Thuret R, Abdollah F, Lughezzani G, Schmitges J, Tian Z (2011). Age-adjusted incidence, mortality, and survival rates of stage-specific renal cell carcinoma in North America: a trend analysis. Eur Urol.

[42] Shields M, Tjepkema M (2006). Trends in adult obesity. Health Rep.

[43] Hu J, Mao Y, White K, Canadian Cancer Registries Epidemiology Research Group (2003) Overweight and obesity in adults and risk of renal cell carcinoma in Canada. Soz Praventivmed 48(3):178–185 10.1007/s00038-003-2046-2 12891869

[44] Pan SY, DesMeules M, Morrison H, Wen SW, Canadian Cancer Registries Epidemiology Research Group (2006) Obesity, high energy intake, lack of physical activity, and the risk of kidney cancer. Cancer Epidemiol Biomarkers Prev 15(12):2453–2460 10.1158/1055-9965.EPI-06-0616 17164370

[45] McGuire BB, Fitzpatrick JM (2011). BMI and the risk of renal cell carcinoma. Curr Opin Urol.

[46] Purdue MP, Moore LE, Merino MJ, Boffetta P, Colt JS, Schwartz KL (2013). An investigation of risk factors for renal cell carcinoma by histologic subtype in two case–control studies. Int J Cancer.

[47] Joffres MR, Hamet P, Rabkin SW, Gelskey D, Hogan K, Fodor G (1992). Prevalence, control and awareness of high blood pressure among Canadian adults. Canadian Heart Health Surveys Research Group. CMAJ.

[48] Public Health Agency of Canada (2010) Report from the Canadian chronic disease surveillance system: hypertension in Canada, 2010. Public Health Agency of Canada, Ottawa

[49] Colt JS, Schwartz K, Graubard BI, Davis F, Ruterbusch J, DiGaetano R (2011). Hypertension and risk of renal cell carcinoma among white and black Americans. Epidemiology.

[50] Reid J, Hammond D (2011). Tobacco use in Canada: patterns and trends.

[51] McLaughlin J, Lipworth L, Tarone R, Blot W, Schottenfeld D, Fraumeni JJ (2006). Renal cancer. Cancer epidemiology and prevention.

[52] Hunt JD, van der Hel OL, McMillan GP, Boffetta P, Brennan P (2005). Renal cell carcinoma in relation to cigarette smoking: meta-analysis of 24 studies. Int J Cancer.

[53] Gandini S, Botteri E, Iodice S, Boniol M, Lowenfels AB, Maisonneuve P (2008). Tobacco smoking and cancer: a meta-analysis. Int J Cancer.

[54] Hu J, Ugnat AM (2005). Canadian Cancer registries epidemiology research G. Active and passive smoking and risk of renal cell carcinoma in Canada. Eur J Cancer.

[55] Theis RP, Dolwick Grieb SM, Burr D, Siddiqui T, Asal NR (2008). Smoking, environmental tobacco smoke, and risk of renal cell cancer: a population-based case–control study. BMC Cancer.

[56] Reid J, Hammond D, Burkhalter R, Rynard V, Ahmed R (2013). Tobacco use in Canada: patterns and trends.

[57] Gotay CC, Katzmarzyk PT, Janssen I, Dawson MY, Aminoltejari K, Bartley NL (2013). Updating the Canadian obesity maps: an epidemic in progress. Can J Public Health.

[58] World Cancer Research Fund/American Institute for Cancer Research (2007) Food, nutrition, physical activity and the prevention of cancer: a global perspective. AICR, Washington, DC

[59] Grosz A (2004). A preliminary geochemical map for arsenic in surficial materials of Canada and the United States. Appl Geochem.

[60] Hollingsworth JM, Miller DC, Daignault S, Hollenbeck BK (2006). Rising incidence of small renal masses: a need to reassess treatment effect. J Natl Cancer Inst.

[61] CancerCare Manitoba (2010) Annual statistical report: Department of Epidemiology & Cancer Registry, 2010

[62] Pantuck AJ, Zisman A, Belldegrun AS (2001). The changing natural history of renal cell carcinoma. J Urol.

[63] Howlader N, Noone AM, Krapcho M, Garshell J, Neyman N, Altekruse SF et al (2013) SEER cancer statistics review, 1975–2010. National Cancer Institute. Bethesda, MD. http://seer.cancer.gov/csr/1975_2010/ based on November 2012 SEER data submission, posted to the SEER web site

[64] Brenner H, Francisci S, de Angelis R, Marcos-Gragera R, Verdecchia A, Gatta G (2009). Long-term survival expectations of cancer patients in Europe in 2000–2002. Eur J Cancer.

[65] Beebe-Dimmer JL, Colt JS, Ruterbusch JJ, Keele GR, Purdue MP, Wacholder S (2012). Body mass index and renal cell cancer: the influence of race and sex. Epidemiology.

[66] De P, Dryer D, Otterstatter MC, Semenciw R (2013). Canadian trends in liver cancer: a brief clinical and epidemiologic overview. Curr Oncol.

[67] Otterstatter MC, Brierley JD, De P, Ellison LF, Macintyre M, Marrett LD (2012). Esophageal cancer in Canada: trends according to morphology and anatomical location. Can J Gastroenterol.

[68] Peterson E, De P, Nuttall R (2012). BMI, diet and female reproductive factors as risks for thyroid cancer: a systematic review. PLoS One.

[69] Chow WH, Gridley G, Fraumeni JF, Jarvholm B (2000). Obesity, hypertension, and the risk of kidney cancer in men. N Engl J Med.

[70] Weikert S, Boeing H, Pischon T, Weikert C, Olsen A, Tjonneland A (2008). Blood pressure and risk of renal cell carcinoma in the European prospective investigation into cancer and nutrition. Am J Epidemiol.

[71] Luciani LG, Cestari R, Tallarigo C (2000). Incidental renal cell carcinoma-age and stage characterization and clinical implications: study of 1092 patients (1982–1997). Urology.

